# Affective communication: a mixed method investigation into COVID-19 outbreak communication using the Taiwanese government Facebook page

**DOI:** 10.1177/17579759211003539

**Published:** 2021-05-13

**Authors:** Chia Yu Lien, Yun-Hsuan Wu

**Affiliations:** 1 Washington University in Saint Louis, Saint Louis, Missouri, United States; 2 China Medical University, Taichung, Taiwan

**Keywords:** COVID-19, Taiwan, outbreak communication, Facebook

## Abstract

The COVID-19 outbreak has created an unprecedented challenge for governments to convey information to the public, and social media has become a critical method of COVID-19 communication in Taiwan.

**Objectives:**

This study examines a total of 1128 Facebook posts published by Taiwan’s principal health authority from December 1, 2019 to May 31, 2020.

**Methods:**

Using both qualitative and quantitative approaches, this study investigates strategies used by the Taiwan government to communicate the COVID-19 outbreak and public responses toward these strategies.

**Result:**

Novel uses of Facebook posts on outbreak communication were identified, including solidarity, reviews of actions, press conferences, and the use of animal and cartoon images. Quantitative results showed that the public responded significantly more frequently to messages generating positive affects, such as posts that reviewed government actions and public efforts; posts that expressed thanks, approval, or comradeship; and posts that paired text with photographs of frontline workers or cute animals.

**Conclusion:**

These results suggest that, amid a disease outbreak, the public not only look for updated situations and guidelines but also for affective affirmation from government agencies.

## Introduction

The COVID-19 crisis has created an unprecedented challenge for governments to convey information to the public. Social media (e.g. Facebook and Twitter) has played an ever-increasing role in health communication (1). Building on Web 2.0 technologies, social media provides government platforms to disseminate timely information (2,3). In the past two decades, the Taiwan government has increasingly used social media for policy communication (4). Understanding social media used by the Taiwan government provides a good opportunity to examine the relationship between COVID-19 outbreak communication and public responses.

Studies have begun to examine the role of social media in infectious disease communication over the past decade. Using the thematic analysis method, these studies identified themes in manifest content published by governmental agencies to communicate a disease outbreak (5). For example, Ding and Zhang (6) identified six themes from the investigation of 163 posts published on Facebook and Twitter by the US Centers for Disease Control and Prevention (CDC) and Department of Health and Human Services (DHHS) in response to the 2009 H1N1 outbreak: case updates, policies and guidelines, prevention topics, official actions and efforts, general information, and scientific research. Kim and Liu (7) compared messages posted by governments and private sectors in response to the 2009 flu outbreak and reported that governmental agencies are more likely to update situations and to provide instructive information. Likewise, Wong *et al*. (8) analyzed 1648 tweets made by US local health departments in response to the 2014 Ebola epidemic and proposed four themes that are more likely to be published: information giving, preparation, news updates, and event promotion.

These studies provide valuable insight into the message strategies used by governmental agencies during a disease outbreak, yet how the public would respond to these strategies has not been fully addressed. Social media could serve as platforms for information dissemination only when users respond to the messages. On Facebook, for example, messages that users like, comment on, and share will be broadcast to their network of friends (9,10). Previous research on Facebook has shown that different message strategies generate different responses. Kim and Yang (11), for example, examined 600 Facebook posts published by 20 companies and found that users often liked posts paired with multimedia elements, while they shared posts both with and without these elements. They also found that *like* is affective, while *share* is either affective or cognitive, or a combination of both. Finally, they noted that *like* indicated the lowest level of commitment, while *share* indicated the highest. A later study (12) has supported their conclusion that *like* and *share* represent different affects and commitments.

Limited existing research has shown that the public might respond differently to various types of messages used by governmental agencies during an infectious disease outbreak. Studying the Singapore 2016 Zika outbreak, Vijaykumar *et al*. (13) found that the public most often liked government Facebook messages on investigation, while sharing messages on news updates. Lwin *et al*. (14) found that the public most often liked government Facebook messages that emphasized self-efficacy, while they shared messages about risk, uncertainty reduction, and reassurance. Additionally, Lwin *et al*. reported that the public was more likely to react to posts made before the outbreak then, after that, the frequency of both likes and shares decreased. The results indicated that the public ceased to disseminate even important information that was familiar to them.

So far, research on infectious disease communication has tended to examine strategies in terms of message content. However, studies on government communication showed that both message content and multimedia formats (photos, videos, etc.) impacted public engagement and information dissemination. Bonsón *et al*. (15) investigated the use of Facebook by governmental agencies in 15 different countries and suggested that text messages paired with photographs effectively increased public engagements. Similar findings are demonstrated by Lev-On and Steinfeld (16), who studied Facebook use by the Israeli governments. To our knowledge, no studies have investigated the effectiveness of multimedia used by governmental agencies to communicate an infectious disease outbreak. This phenomenon is worth investigating since it provides insight into strategies that sustain public engagement with important infectious disease information.

The current study investigates the strategies (contents and multimedia formats) used by the Taiwan government to communicate COVID-19 outbreak information, and public responses toward these strategies. Taiwanese officials confirmed the first COVID-19 cases on January 21, 2020. Since December 31, 2019, the Taiwan government has implemented more than 100 policies and actions to contain the outbreak (17). Social media, particularly Facebook, has become a critical locus of COVID-19 communication (18). However, strategies used by governmental agencies to communicate COVID-19 information on this platform have not been studied. To address this issue, the study asks three questions: (i) what were the characteristics of, and the public responses toward, governmental Facebook posts about COVID-19 in Taiwan; (ii) what content and multimedia strategies did the Taiwan government use to communicate information concerning the COVID-19 outbreak; and (iii) what were the public responses to these different strategies? The findings of this study will increase current knowledge about the effectiveness of infectious disease communication.

## Method

### Data collection

To better understand the government’s COVID-19 communication, this study examined all Facebook posts made by the Taiwan Ministry of Health and Welfare (MOHW) from December 1, 2019 to May 31, 2020. The posts were collected for this period as it marked the first post published by the MOHW in December 2019 up to the current date. The MOHW Facebook page was selected for two reasons: First, the MOHW is the principal health authority of Taiwan and is responsible for COVID-19 prevention and information communication. Second, the MOHW is the most-followed governmental health agency, followed by more than 600,000 people as of May 31, 2020. An Excel spreadsheet was created with all posts downloaded from the MOHW Facebook page, which included text content, multimedia formats (images, videos, etc.), the published date of each post, and the number of *likes* and the number of *shares* associated with each post.

### Post analysis

All MOHW Facebook posts were first coded based on whether COVID-19 messages were present. COVID-19-related posts were further analyzed using a combination of inductive and deductive coding as proposed by Bernard *et al*. (19). According to Bernard *et al*, this coding started with themes derived from previous studies and added more themes and subthemes as the process continued. This method was preferred because it allowed us to compare our findings with previous studies on infectious disease communication. Specifically, to analyze post content, we drew on themes of preparation, news updates, event promotion, and information giving from Wong *et al*.’s article (8). To analyze post multimedia formats, we drew on the formats of videos, photographs, links, and texts from Bonsón *et al*.’s article (15). In Bonsón *et al*.’s article, texts paired with photos, links or videos were counted as for the latter. Only posts without all these multimedia forms were counted as texts.

Two researchers then used the grounded theory proposed by Bernard (19) to read each post line by line and note the keywords. The researchers pulled posts with similar keywords together and discussed how each group of posts might be related to the themes and formats derived from previous studies. From this process, we renamed ‘event promotion’, which is defined by Wong *et al*. (8) as a ‘physical or virtual platform to deliver information’ to ‘tech promotion’ and grouped it under ‘preparation’, which included information about actions implemented by governmental agencies to contain the disease. We added new themes of recommendation, solidarity, press conferences, and reviews of actions, that had not been mentioned in Wong’s article. In multimedia analysis, we deleted the category of ‘link’ since it was not used by the MOHW. We added two new formats, animal and comic, that had not been mentioned in Bonsón *et al*.’s article (15) but repeatedly appeared in our data. A codebook was then developed using NVivo 10 QSR International software.

### Public response analysis

We collected information about the number of *likes* and *shares* based on identified contents and multimedia formats and reported them by mean and standard deviation. In order to further explore the public response regarding post content and multimedia, we focused on COVID-19 related posts and used one-way ANOVA to determine whether the average number of *likes* and *shares* across various types of content, as well as multimedia, were different. Additionally, we implemented Bonferroni post hoc analyses to determine which of the content and multimedia type differed significantly on *likes* and *shares*. All analyses were carried out with the STATA SE statistical package, version 14.

## Results

Table 1 describes the monthly conditions of the MOHW Facebook posts and the public responses. The first COVID-19 post was published on December 31, 2019. From December 1, 2019 to May 31, 2020, a total of 1128 posts were collected for analysis, including those created by the MOHW and those created by other government agencies that were then shared by the MOHW. Out of these posts, 925 (82%) were COVID-19-related. From December to February, there was a sharp decline in the number of non-COVID-19-related posts, and a sharp increase in COVID-19-related posts. After this, the number of COVID-19-related posts plateaued at 7–8 posts published by the MOHW each day.

**Table 1.** Descriptions of MOHW Facebook posts and the public responses.

*Month*	Posts not about COVID-19 N (%)	Posts about COVID-19 N (%)	Total posts N	Average likes of COVID-19 posts	Average likes of non-COVID-19 posts	Average shares of COVID-19 posts	Average shares of non-COVID-19 posts
December, 2019	84 (98.82)	1 (1.18)	85	1800	732.64	1200	338.38
January, 2020	62 (46.27)	72 (53.73)	134	1977.03	1209.85	787.82	533.13
February, 2020	6 (2.64)	211 (97.36)	227	8606.99	4583.33	600.72	533.33
March, 2020	19 (7.98)	219 (92.02)	238	7615.52	5231.21	622.05	436.16
April, 2020	22 (9.44)	211 (90.56)	233	13081.04	11386.36	729.55	899.59
May, 2020	11 (4.95)	211 (95.05)	222	12540.47	5671.00	464.19	226.00

The public response showed that COVID-19 posts had outpaced non-COVID-19 posts in the number of *likes* and *shares* received, with the exception of the number of *shares* in April 2020. The number of *likes* COVID-19 posts received had increased from January, with nearly four times as many *likes* in February and seven times as many in April. Meanwhile, the number of *shares* COVID-19 posts received peaked in December, then declined in the following months.

### Analysis of government COVID-19 posts

Table 2 shows the results of a thematic analysis on post contents. Of 925 COVID-19 posts, 27.24% were news updates, 24.21% were recommendations, 23.46% were on preparation, 10.81% were on solidarity, 8.11% were on information-giving, 4.43% were on press conferences, and 1.73% were on reviews of governmental actions. The intercoder reliability was calculated using Cohen’s Kappa coefficient. The coefficients were excellent (0.75–1.00) for all thematic categories, with the exception of information-giving (0.40–0.75), which overlapped with news updates, behavioral recommendations, and preparation.

**Table 2.** Content analysis on government COVID-19 posts

Theme categories	Description	Examples	n	%
**News update**	**New information on COVID-19 disease outbreak**		**252**	**27.24**
Case reports	Description of current domestic and international COVID-19 cases	The Central Epidemic Command Center (CECC) announced that to date (5/29), there have been a total of 442 confirmed COVID-19 cases. Among these cases, 351 were imported and 55 were local	120	12.97
New cases	Reports on new confirmed domestic cases	The newest confirmed case is a 30-year-old woman who worked in the United Kingdom. She reported symptoms upon arrival. She was tested and hospitalized. We will investigate the contacts on the plane	82	8.86
Change in status	Shift in COVID-19 cases regarding isolation and hospitalization	There is a total of 379 confirmed COVID-19 cases: 67 were released from isolation, and the rest remain in isolation in hospital	50	5.41
**Information giving**	**General information on COVID-19**		**75**	**8.11**
Provides knowledge	Information on disease mechanism, diagnosis, and treatment	National Taiwan University public health professor Dr. Lin and his team found out that transmissibility of COVID-19 was higher among those whose exposure to index cases started within five days of symptom onset	44	4.76
Dispelling myths	Statement to clarify misinformation	‘Theaflavin,’ which is found in black tea or pu’er tea, cannot help to fight coronavirus. Do not believe the rumors and avoid passing them on to friends or family	31	3.35
**Preparation**	**New information on policies implemented against COVID-19**		**217**	**23.46**
Cross-border preparation	Information on border control policies and actions	Taiwan bans airline passenger transit through the country starting on March 24 until April 7 to prevent the spread of COVID-19 via air transport	43	4.65
Domestic preparation	Information on domestic policies and actions	All inbound travelers should fill in the Quarantine System form upon entry. Those living with older adults, children, people with chronic illnesses, or those without a separate room (including a separate bathroom) are required to stay at a quarantine hotel	64	6.92
Ban lifting	Information on ban lifting	Since the risk of community transmission is low, CECC decided to reduce restrictions on psychiatric hospital visits	7	0.76
Resource allocation	Information on financial or material resources	Provisions have been adjusted for the name-based mask distribution system starting on April 9. Masks can be bought every 14 days — nine adult masks or ten child masks	89	9.62
Tech promotion	Information on digital resources	We collaborated with HTC DeepQ and Line to develop the Line Bot, which enables people in quarantine to ask questions and report their health status to civil servants	14	1.51
**Recommendation**	**Behavioral guidance and warnings to prevent COVID-19**		**224**	**24.21**
Recommended behavioral changes	Actions one can take to prevent the spread of COVID-19	Keep social distancing with others. Maintain a 1.5-meter distance in indoor environments. Maintain a 1-meter distance in outdoor environments. Wear face masks on public transportation	191	20.65
Warning against misconduct	Statement to warn and fine people for misconduct	Violation of regulations on quarantine / isolation are subjected to a penalty fine of NT 1,000,000 maximum	33	3.57
**Other themes**				
Solidarity	Expressions of thanks, approval, and comradeship	We thank medical workers. Taiwan nurses, we stand with you. More than 170,000 nurses currently work in hospitals, clinics, schools, airports, and other facilities. Please send these nurses a picture or a message	100	10.81
Review of actions	Review of actions taken by government	The virus treats people equally, but in Taiwan, we handled it differently. This video is dedicated to the Taiwanese. We wish you all the best	16	1.73
Press conference	Video of press conference	April 30 COVID-19 press conference will start at 14:00	41	4.43

Posts coded as news updates provided information on international and domestic COVID-19 infection situations, including new confirmed cases, current case reports, and changes in status of isolation and quarantine. These posts also updated the risk of exposure for a specific geographic area. Posts coded as recommendations encouraged certain behaviors, such as handwashing and mask-wearing, and discouraged other behaviors, such as dumping masks and violating quarantine.

Posts on preparation informed policies implemented by governments, including cross-border preparation, such as travel alerts, flight transfer policy, and domestic preparation, such as policies related to quarantine, social gatherings, and testing. They also informed policies lifted in April and May after a period of absence of new cases. Moreover, posts under these themes provided information on resource allocation, such as mask availability and accessibility, and technical support on information and resource access.

Posts coded as solidarity encouraged the public to express their appreciation for frontline workers and show comradeship with their fellow citizens. These posts prompted members of the public to think of themselves not as separate individuals but as part of a wider community, and, in doing so, remember the vulnerability of others. Posts coded as information-giving provided COVID-19-related knowledge on disease transmission, prevention, and treatment. A small number of posts in this category were made to dispel myths and fake news.

Finally, 41 posts were press conferences, which have been held daily since February to update the public on COVID-19 situations, announce governmental preparations, recommend behavioral changes, facilitate solidarity, and to answer questions raised by journalists. Sixteen posts were made to review the actions taken by governmental agencies and the performance of Taiwan regarding the outbreak.

Table 3 shows results for the thematic analysis on post multimedia types. Of all COVID-19 posts, 44.11% were in pure text form, 28.54% in text paired with images of animals (263 with a shiba inu dog), 10.38% with photographs, 10.05% with comics, cartoons, or emojis, and 6.92% with videos. The coefficients were excellent (0.75–1.00) for all multimedia categories.

**Table 3.** Multimedia analysis on government COVID-19 posts.

*Category*	Description	Examples	n	%
Photograph	Posts that include photos.	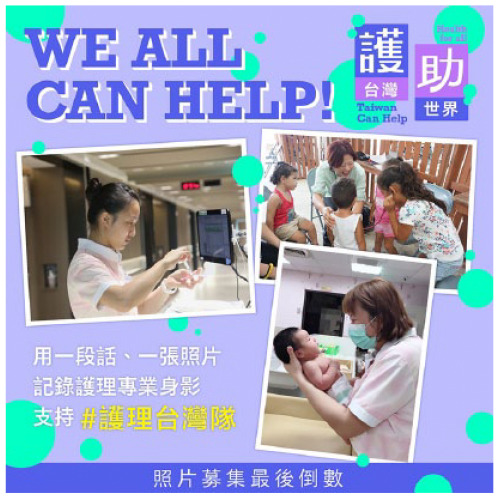 URL: https://www.facebook.com/470265436473213/posts/1551563158343430/	96	10.38
Video	Posts that include videos.	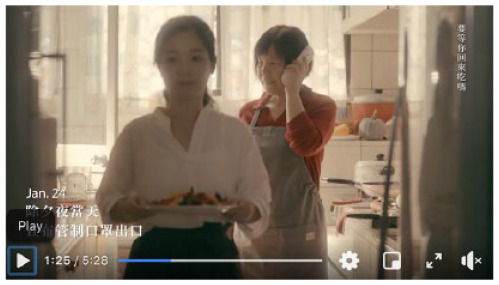 URL: https://www.facebook.com/470265436473213/posts/1569196643246748/	64	6.92
Comic	Posts that include comics, cartoons, or emojis.	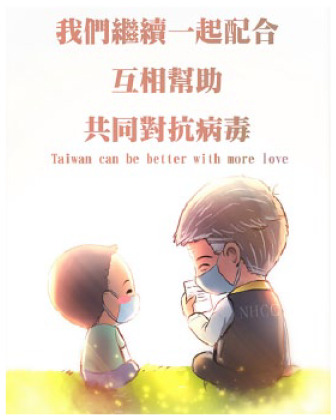 URL: https://www.facebook.com/470265436473213/posts/1530064037160009/	93	10.05
Animals	Posts that include animals.	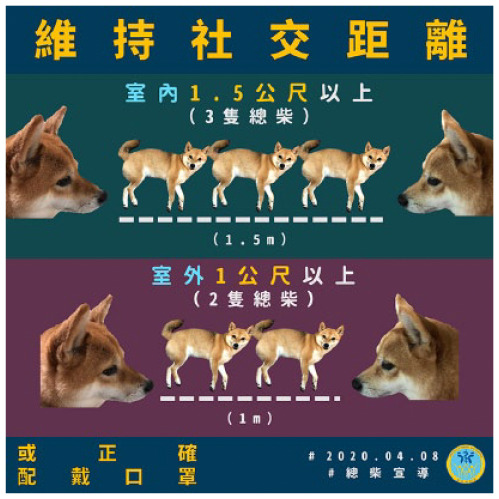 URL: https://www.facebook.com/mohw.gov.tw/photos/a.484593545040402/1530089737157439/	264	28.54
Pure text	Posts with a plain background.	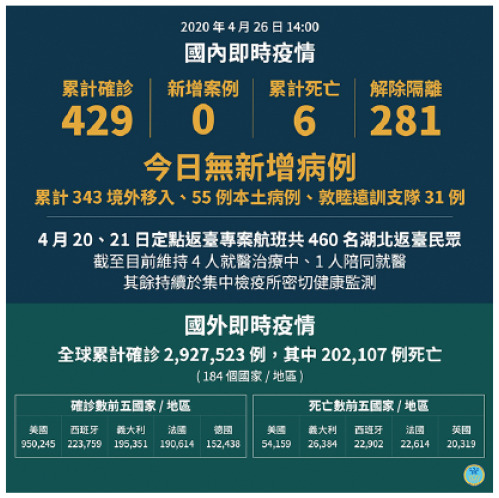 URL: https://www.facebook.com/470265436473213/posts/1547279608771785/	408	44.11

### Public responses to the government posts

On average, each COVID-19 post received 9766.66 *likes* and 628.69 *shares*. Table 4 provides results on *likes* and *shares* received by each content type and multimedia format. Different content types received significantly different numbers of *likes* (*p* < 0.05) and *shares* (*p* < 0.05). Post hoc analyses further showed that, compared to other types, posts that reviewed actions taken by the government received the highest number of *likes* and *shares* (*p* < 0.005). Posts on solidarity received a significant number of *likes* compared to news updates (*p* = 0.007), recommendations (*p* < 0.001), preparation (*p* < 0.001), information-giving (*p* < 0.001), and press conferences (*p* < 0.001). Content providing information received a significant number of *shares* compared to posts on press conferences (*p* = 0.049).

**Table 4.** Public responses to the government posts.

Responses	Like	Share
	*Mean (SD)*	Mean (SD)
Content		
News update	11136.77 (15136.64)	541.45 (769.63)
Information-giving	5497.95 (7134.76)	853.03 (1511.18)
Preparation	7661.15 (10030.29)	754.35 (1358.65)
Recommendation	7727.62 (9068.30)	576.55 (1141.21)
Solidarity	17421.00 (26887.39)	410.11 (421.60)
Press conference	4037.00 (2365.83)	67.88 (44.71)
Review of actions	32356.25 (40727.08)	2814.69 (6244.12)
p-Value	<0.01	<0.01
Multimedia		
Animal	11394.05 (12500.22)	631.27 (1105.76)
Comic	9190.66 (9609.94)	571.20 (919.97)
Photograph	18735.29 (31949.10)	782.76 (1252.77)
Pure text	7371.49 (10993.70)	616.42 (1141.36)
Video	5754.92 (12335.70)	557.95 (3165.99)
p-Value	<0.01	0.81

Different multimedia formats received significantly different numbers of *likes* (*p* < 0.05), but not *shares* (*p* > 0.05). Posts including photos received the highest number of *likes* and *shares*, followed by posts including animals. Post hoc analyses showed that posts including photos received a significantly higher number of *likes* compared to all other formats (*p* < 0.001). Posts including animals received a greater number of *likes* compared to posts of pure text (*p* = 0.007).

## Discussion

This study examined the characteristics and effectiveness of social media use by the Taiwan government agency to communicate various COVID-19 issues. The findings show that the Taiwan government agency has actively used Facebook to communicate the COVID-19 outbreak. Parallel to previous studies (14), the findings show that the public increased their *shares* of infectious disease information in the pre-outbreak phase, then decreased them after they gained familiarity with the disease information. However, in contrast to previous studies (14), we found that people in Taiwan increased their *likes* of governmental posts after the pre-outbreak phase, demonstrating that the government had generated sustained public attention toward the COVID-19 situation.

To investigate strategies applied by the government agency to sustain public responses, this study analyzed thematic categories of content and multimedia of COVID-19 posts and the public responses to each category. Most posts fell into the thematic categories of news updates, behavioral recommendations, and government preparations, which provided information on the incidence, spread, and containment of the outbreak. These findings align with the analysis of previous research on government infectious disease communication that has identified the use of social media by governmental agencies to provide information and advice (20,21). Our study also identified thematic categories that are rarely mentioned, including posts that encouraged expressions of thanks, approval and comradeship, and posts that reviewed efforts made by government agencies and the public. Rather than providing new outbreak information, these categories offer emotional support and facilitate solidarity between citizens and the country.

It was found that people were more inclined to *like* posts on review of actions, solidarity, and news updates, and *share* posts on review of actions, information giving, and preparation. The findings regarding *likes* provide new insight into the needs of the public during a disease outbreak, indicating that the public sought affective affirmation from the government during the crisis, and they were encouraged by efforts made by the government and themselves to contain the disease. The findings on *shares* confirm the importance of affective affirmation. Meanwhile, the findings regarding *shares* also confirm that the public deemed information that promoted their knowledge and preparation around COVID-19 to be important (22). The discrepancy between *like* and *share* parallels Kim’s finding (7) that *likes* indicate an affective response while *shares* indicate either an affective or a cognitive response.

Five thematic categories were identified from the multimedia of the posts. Half of the posts were purely text-based, and some posts contained text paired with photographs of frontline workers or videos. All these formats are common in governmental use of social media (15,16). However, we also identified that the Taiwan MOHW paired more than one-third of COVID-19 information with animal or comic images, unseen in previous research on government use of social media. We called this strategy ‘playing cute.’ In this context, ‘cuteness’ is defined as having the appearance of an infant or animal (i.e., a small body, round eyes, and a chubby face) (23,24). Images of cuteness are commonly used in both the political and apolitical spheres in Taiwan (24). Cultural studies research has shown that cute images or objects create a more personal feeling for the reader. In Taiwan, these images and objects have been demonstrated to be an effective tool in advertisements (24).

Our results found that people mostly *liked* COVID-19 text paired with photographs, animals, and comics, while they *shared* posts with text paired with photographs and animals, along with text-based posts. They were least inclined to *like* or *share* videos published by governmental agencies. On social media, multimedia formats have been demonstrated to affect public engagement significantly (25,26). Photographs of frontline workers, such as governmental officials, civil servants and medical staff are mostly engaging, which is parallel with studies showing that the public often responds to photographs of events and news relevant to them (15,26). Meanwhile, our findings show the effectiveness of ‘playing cute’ in infectious disease communication in Taiwan: COVID-19 posts containing text paired with images of cute dogs were more likely to be *liked* and *shared*. The popularity of text paired with workers’ photos and cute animals supports our finding that posts generating positive affects are more likely to be disseminated, indicating that people look for calming messages from the government during a disease outbreak.

Affect has been identified as a driving force for online information dissemination (27,28). Limited existing research shows that some government agencies have incorporated reassuring messages as part of infectious disease communication. Studying the responses toward the 2009 H1N1 virus, Liu and Kim (29) observed that compared with corporate organizations, government agencies (US CDC, US DHHS, and the WHO) were less likely to incorporate affective messages, yet when they did, they often sent messages of sympathy and awareness, while corporate organizations sent messages of fear. Likewise, Lwin *et al*. (14) reported that the Singapore government incorporated calming and thanking messages to remove uncertainty and fear surrounding the threat of Zika, which attracted a great number of *likes* and *shares* in the pre-outbreak phase. Our results add that affective affirmation plays a key role in sustaining public attention toward infectious disease messages in the post-outbreak phase: though the public decreasingly shared COVID-19 posts after they gained familiarity with the information, they increasingly liked those posts conveying positive affects. We also observed that in addition to reassuring contents (reviewing actions taken by the government and the public, and expressing solidarity), reassuring or soothing images (photographs of frontline workers and cute animals) can facilitate infectious disease communication.

Based on our findings, we suggest that government agencies pay attention to the role of affect in policy communication in different phases of a pandemic. We also suggest that government agencies incorporate multimedia formats to facilitate information dissemination. Finally, we are aware that people of different cultures may perceive and react to similar messages differently. The effectiveness of using ‘playing cute’ by the MOHW, for example, reflects not how a government agency creates a new strategy for communication, but utilizes an already existing, culturally effective one. Therefore, we suggest that government agencies investigate and integrate local culture to strengthen the coordination of the public. This study has several limitations. First, while this study focused on infectious disease communication in Taiwan, future research should consider studying the same topic in different countries to compare the effectiveness of affective affirmation in information dissemination. Second, while this study used *likes* and *shares* to estimate the spread of COVID-19 information, further study on social networks should be carried out to determine the actual distribution of messages. Third, we did not analyze the content of reactions using comments. More in-depth content analysis of comments is needed to reveal the interactions and discussions between the public and the government.

## Conclusion

COVID-19 had infected over one million people globally by the end of May 2020. In Taiwan, as of May 31st, only 442 people in a population of 23 million had been identified as being infected, and only 7 of these people died (30). Taiwan’s performance on COVID-19 containment was a result of the government’s multiple policies and actions, such as border control measures, travel restrictions, and various resource allocations. However, without an effective tool for communication, these actions might have caused confusion and frustration. This research provides insight into the strategies applied by the Taiwan principal health agency to communicate about the disease outbreak. It is among the few studies to analyze the impact of the contents of Facebook posts and the use of multimedia formats in posts on the effectiveness of information dissemination. On social media, a piece of information always needs to compete with other messages for engagement. Our results provide insight into strategies that can be used by health departments to inform the public about an emerging infectious disease.
